# Another explanation for the low allergy rate in the rural Alpine foothills

**DOI:** 10.1186/1476-7961-3-7

**Published:** 2005-06-05

**Authors:** Matthias Wjst

**Affiliations:** 1 Institut für Epidemiologie GSF – Forschungszentrum für Umwelt und Gesundheit Ingolstädter Landstrasse 1 D-85758 Neuherberg / Munich Germany

## Abstract

A low allergy rate in coal and wood heated homes has been described in the small villages in the Alpine foothills and subsequently found to be associated with the farming environment. This was interpreted within the framework of the hygiene hypothesis but there are also alternative explanations. Lower air pollution could be one reason, which is, however, unlikely since the differences between the Bavarian countryside and the Munich municipal area were only weak. There could be genetic differences between the urban and rural population by previous isolation or by self-selection. The potential drop-out of allergy genes, however, will also not explain the absent increase of allergies in two generations. More likely, other lifestyle factors are important. Dietary habits are different in farmers and a less frequent vitamin D supplementation of newborns (otherwise expected to be allergy promoting) has been shown recently. The underlying cause for the "non-allergic farm child" remains speculative until the transfer of any farm-associated factor is leading to a similar risk reduction in the general population.

## Introduction

Allergy prevalence has been on the rise worldwide and nearly hundred years after coining "Allergie" in the "Münchner Medizinische Wochenschrift" [1] the causal risk factors are still unknown.

At the end of the 1980s, air pollution related effects were thought to be responsible for the allergy epidemic. It turned out, however, that at least traffic related combustion was not the main culprit in the Munich municipal area, neither based on the inner city distribution of pollutants [2] nor by comparison with a control region in Upper Bavaria. In this study, located in the South of Munich on the Alpine foothills, I examined nearly two thousand fourth-grade children between October 1989 and July 1990 in more than 50 villages. I already noted at that time a relationship between the farm odour in some of the small classrooms and the nearly absence of any positive skin prick test (the "Ostallgäu" phenomenon). A protective effect of coal heating was eventually published six years later [3] but received little attention as the public interest focused mainly on East- and West German air pollution differences [4]. It was only in 1999 where a long series of studies in the farming environment started [5-12] which lead to the current version of the hygiene hypothesis that allergy develops where the natural high endotoxin level on farms is absent. Endotoxin has already been described in a study in 2000 as the main component protecting against allergic sensitisation [13].

## Problems with the hygiene hypothesis

The hygiene hypothesis is based on the the initial observation of "unhygienic" siblings by Golding and Peters 1986 [14]. After more than one decade of research [15], however, Strachan concluded that "an inverse association between infection and allergy has not been confirmed directly by epidemiological studies. The available data are either inconsistent or inconclusive" [16]. This view is supported by several other authors [17-19] as the adaptive immune system "with an array of potential interactions .... is reduced to a single level" [20]. Although even a patent has been filed on components of stable air to treat allergy [21], a task force of the European Academy of Allergology and Clinical Immunology (EAACI) arrived at the opinion that "there is no recently published evidence in favour of a clinical use of so-called bacterial extracts against asthma and allergic diseases." [22]

During the discussion of factors related to hygiene it seemed to be largely neglected, that (viral) infection may even enhance allergic disease [23]. Also the inverse association of hepatitis antibodies and allergy found in Italian military students [24] has not been reproduced in consecutive studies [25-27]. The protection against allergic disease by mycobacteria [29] could also not be reproduced in the following dozen studies [28]. The support for the hygiene hypothesis therefore remains weak.

Unfortunately, all farming studies are based on observational and retrospective data given rise to concerns not mentioned in previous reviews [30,31]. The transition of a farming society into the industrial age neither coincides with the main peak of the allergy prevalence in Western countries nor does it match the geographical distribution of the disease.

## Is endotoxin to blame?

Although there are well-designed studies describing the immunological action of endotoxin [32-35] there are no quantitative data in humans how the nanogram exposure on the pulmonary epithelium will supersede the gram-wise exposure on the gut mucosa. The number of bacteria on the human body's surface is more than 10 times greater than all his somatic cells [36]. Even if N-acetyl-muramic acid is found to be significantly higher in dust from farm children's mattresses (+20% [37]) or endotoxin units are being increased (+66% [10]), is is unclear whether this has any biological meaning [38]. There are many reasons why dust deposition on the floor may not be equal to effective exposure as this involves inhalation, deposition, uptake, processing, preservation and target delivery. In the only study available so far, both asthmatic and non asthmatic probands had the same LPS concentrations in their bronchoalveolar lavage [39].

Even if we assume a relevant target exposure, there are effective mechanism to counteract endotoxin [40,41]. Dose and timing [42], even the origin from different bacteria [43,44] as well as host characteristics [38,45] are being important. Lipopolysaccharides from some bacteria may induce even a Th2 type response [44] where the induction of sensitization is an allergen-specific phenomenon that can not be simply attributed to endotoxin [46]. Epidemiological effects of LPS in dust are often found with extremes of the distribution only, either not significant [47], marginally significant [13,6,48-50], non-linear [10], heterogeneous [51] or even in the opposite direction [52-54].

The main contradiction [55], however, stems from the fact that farming is a frequent risk factor for allergy [56] and asthma [57,58]. This might be the explanation why some studies do not find any association between farming and sensitization [59,60] or even opposite results [61].

Research into the biology of endotoxin had many unexpected turns and "has engendered immense curiosity over the years" according to one of its pioneers [41,62]. "Why should diminished exposure to microrganism result in inadequate priming of T regulatory cells?" [63]. Any different LPS exposure effect in early life than later on as suggested by Martinez [64] is contradicted by studies where inhalation of LPS induces airway inflammatory response and wheezing [65-69]. This airway response was dose-dependent in both, healthy and asthmatic subjects [65], genetically determined [70-72] and may be enhanced by concomitant inhalation of allergen challenge [73]. It is therefore not unexpected that endotoxin exposure is still the main determinant of lung function decline in farmers [74,75].

## Are other bacterial components relevant?

With the ubiquitous occurrence of LPS, its association also to non-farm settings [10], or other household factors [76,77] the situation is far from being clear. There might be effects by other bacterial products [37,78] but there are even considerable doubts if bacterial co-factors are responsible for the observed effects. The largest study concluded that "environmental changes affecting the whole of society have promoted an increase in asthma, allergic rhinitis and eczema in both farming and non-farming environments ... whereas the protective effect of growing up on a farm on the risk of asthma appears to be a fairly recent phenomenon" [79]. Similar conclusions are reported in the second largest study where "the percentage of subjects with symptoms of rhinitis or allergic sensitization was generally lower in subjects who had lived on a farm than in other subjects but the difference was significant only in subjects born after 1961" [80]. In addition also a study from Switzerland reported only a very recent increase of allergy in children from non-farming households [7]. If we assume that the bacterial universe did not undergo a major change since 1961, direct bateria-related effects are not very likely.

## What else could explain the "non-allergic farm child" effect?

Lower air pollution by industry or car traffic could be one reason. Unfortunately, this explanation is rather unlikely as the absolute difference between the Upper Bavarian countryside and the Munich municipal area was weak [3].

Second, there might be a self-selection mechanism leading to the drop-out of allergic people, otherwise known in epidemiology as "healthy worker" effect. This phenomenon can hardly explain the absent increase of allergies during the last generation [80].

As there is a clear genetic influence on the development of allergy [81,82] there might be different genes and variants in farmers due to previous isolation. This may be assumed from the unexpected finding of longer linkage disequilibrium blocks in a recent comparison of rural and urban communities [83]. Again, this observation does not explain the recent generational increase although we have argued earlier that the reduction of newborn respiratory mortality by antibiotics may have changed our gene pool [84]. Also other environmental exposure may influence the gene pool. It could be shown recently that elevated levels of folic acid during the periconceptional period could select human embryos that carry a mutant MTHFR allele (with adverse effect on later vascular disease) [85]. Any differential exposure in farmers might therefore be important on their particular genetic background.

Fourth, the socioeconomic situation in the Alpine farmers is different compared to the major cities. There might be a lower vaccination rate although there is no evidence that early vaccination can cause later allergy [86-90]. Farmer might use less antibiotics (an effect under extensive research [87,91-94]), however, the antibiotic level in farm dust has been reported to be high [95]. In one study farm children had more siblings, were more likely to be breast-fed and to have pets [96]. In another study farm children had again more siblings, were more likely to have a cat or dog, to experience more serious respiratory infections and less likely to have attended daycare [80]. A higher number of siblings is in favour of the traditional hygiene hypothesis [15] but adjustment for family size did not resolve the farming effect. Less daycare attendance even argues against the hygiene hypothesis [97,98].

## Do dietary factors play a role?

**Figure 1:**
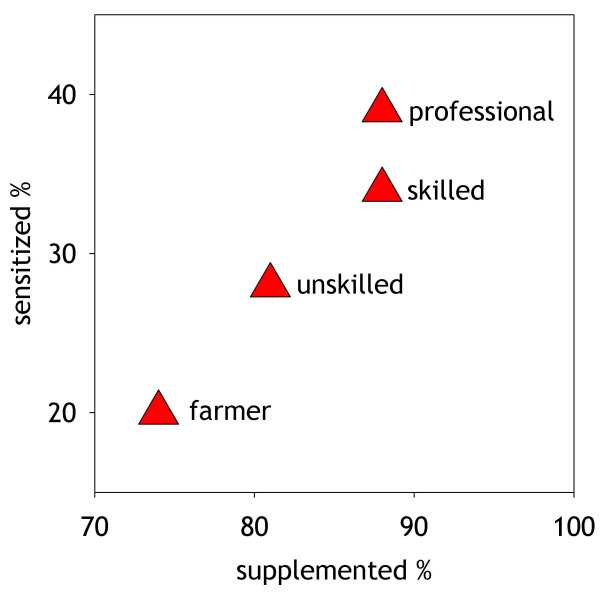
The figure is adapted and drawn from a previous study reported in reference [99]. Included are 10,821 individuals of a Finnish birth cohort, where the percent of individuals with intake of the recommended vitamin D supplementation of 50 μg/day (2000 IU) recorded at the first birthday follow-up in 1967) is plotted against the percent of individuals sensitized against cat, birch, timothy grass or house dust mite at age 31 by profession.

Finally, food and dietary habits may be different in farmers. For example farmers use less aggressive vitamin supplements (Figure 1, [99]). This observation may be important as vitamin D is widely used in the newborn period to prevent rickets [100] although its main metabolite is known to suppresses dendritic cell function resulting in the inability to mount a sufficient Th1 response [101]. Animal [102,103], genetic [104-106] and epidemiological studies [99,107] now support a role in the development of allergy.

This seems to be particular important as the "non-allergic farm child" effect is observed preferentially in a region only after the general introduction of vitamin D supplementation. The upsurge in allergy and asthma prevalence has been identified as a "post-1960s"-epidemic [80,108] which matches exactly with the time point of a general rickets prophylaxis approach in Bavaria [109]. Furthermore, the farm protection was seen mainly found during the first year of life [30,110] where vitamin D supplementation period is now recommended in Bavaria [111,112].

Farmers consume more local foods and less supplements. The protective effect of farm milk could relate to the avoidance of otherwise fortified milk from supermarkets [113]. Although milk is usually not fortified in the Alpine region, nearly all baby foods contain vitamin supplements. An alternative food related hypothesis has been setup for Crohns' disease [114] where the transition of cold food storage could be leading to different bacterial exposure.

## Body height and head circumference, further pieces in the puzzle?

There is also another unpublished observation from our first study 1989 in Upper Bavaria where remote village size was not only associated with less allergic rhinitis but also with decreased body height. An increase in body height is a known effect of vitamin D treatment [115-119]. In a Norwegian study, male farmers were on average 2,3 cm and female famers 1,4 cm smaller (personal communication E. Omenaas 2005 [120]). A more recent German study [121] showed birth weight to be positively associated with later allergic sensitization while in British babies the head circumference was associated with the development of high IgE levels [122-125]. Do vitamin D supplements explain this association?

## Relationship between hygiene and vitamin hypothesis

Both, vitamin and hygiene hypotheses are not mutually exclusive. For example there has been a higher frequency of respiratory infections in vitamin D deficient children [126-129], a phenomenon also found in farming children [80]. On a cellular level it is being known that calcitriol pulsed dendritic cells show a blunted response to LPS [130,131], where LPS pulsed IL-12 response [13,132] can override the otherwise blocking effect of calcitriol (giving possibly farming children a higher capacity to tolerate external vitamin D doses). Similar results have been obtained in human monocytic cells where LPS downregulated vitamin D receptor levels and thus inhibited vitamin D action [133].

## Conclusion

Many of the clinical and epidemiological observations in the farming populations are neither conclusive nor fully understood. Will further studies in the rural Alpine foothills provide the final answer?

## Competing interests

The author(s) declare that they have no competing interests.

## Authors' contributions

The author developed the hypothesis presented here, conducted the literature survey, wrote the paper and approved the final version of the manuscript.

## Funders

My salary is paid by GSF FE 73922.
